# Vitamin D is involved in the effects of the intestinal flora and its related metabolite TMAO on perirenal fat and kidneys in mice with DKD

**DOI:** 10.1038/s41387-024-00297-z

**Published:** 2024-06-10

**Authors:** Xiaodi Zheng, Yuhong Huang, Mengxue Yang, Lulu Jin, Xuemeng Zhang, Rui Zhang, Yueyue Wu, Cuili Yan, Yuan Gao, Miao Zeng, Fei Li, Xue Zhou, Neng Zhang, Jun Liu, Bingbing Zha

**Affiliations:** 1 Department of Endocrinology, Shanghai Fifth People’s Hospital, Fudan University, Shanghai, 200240 China; 2 Community Health Research Center, Fudan University, Shanghai, 200240 China; 3 Pujiang Community Health Service Center, Minhang District, Shanghai, 2011112 China; 4 Department of Infectious Diseases, Shanghai Fifth People’s Hospital, Fudan University, Shanghai, 200240 China; 5 Department of Endocrinology, Affiliated Hospital of Zunyi Medical University, Guizhou, 563000 China

**Keywords:** Type 2 diabetes, Bacteria, Obesity

## Abstract

**Background:**

Vitamin D was shown to directly exert a protective effect on diabetic kidney disease (DKD) in our previous study. However, whether it has an effect on perirenal adipose tissue (PRAT) or the intestinal flora and its metabolites (trimethylamine N-oxide, TMAO) is unclear.

**Methods:**

DKD mice were received different concentrations of 1,25-(OH)_2_D_3_ for 2 weeks. Serum TNF-α levels and TMAO levels were detected. 16S rRNA sequencing was used to analyze gut microbiota. qPCR was used to detect the expression of TLR4, NF-Κb, PGC1α, and UCP-1 in kidney and adipose tissue. Histological changes in kidney and perirenal adipose tissue were observed using HE, PAS, Masson and oil red staining. Immunofluorescence and immunohistochemistry were used to detect the expression of VDR, PGC1α, podocin, and UCP-1 in kidney and adipose tissue. Electron microscopy was used to observe the pathological changes in the kidney. VDR knockout mice were constructed to observe the changes in the gut and adipose tissue, and immunofluorescence and immunohistochemistry were used to detect the expression of UCP-1 and collagen IV in the kidney.

**Results:**

1,25-(OH)_2_D_3_ could improve the dysbiosis of the intestinal flora of mice with DKD, increase the abundance of beneficial bacteria, decrease the abundance of harmful bacteria, reduce the pathological changes in the kidney, reduce fat infiltration, and downregulate the expression of TLR4 and NF-κB in kidneys. The serum TMAO concentration in mice with DKD was significantly higher than that of the control group, and was significantly positively correlated with the urine ACR. In addition, vitamin D stimulated the expression of the surface markers PGC1α, UCP-1 and VDR in the PRAT in DKD mice, and TMAO downregulated the expression of PRAT and renal VDR.

**Conclusions:**

The protective effect of 1,25-(OH)_2_D_3_ in DKD mice may affect the intestinal flora and its related metabolite TMAO on perirenal fat and kidneys.

## Introduction

The gut microbiota is considered as an “organ” that plays important roles in enhancing host immunity, promoting food digestion, regulating intestinal endocrine function, regulating nerve signals, and modulating drug effects and metabolism. This symbiotic relationship between the microbiota and the host ensures the normal development of the host metabolic system. The intestinal microbial metabolites are absorbed by the host act on receptors in organs such as the liver, intestine, brown adipose tissue (BAT), white adipose tissue (WAT), and central nervous system (CNS) and are involved in nutrient synthesis, intestinal motility, and mineral and electrolyte absorption [1].

Dietary choline is metabolized by the gut flora to trimethylamine (TMA) and further oxidized in the liver to trimethylamine N-oxide (TMAO) [2], which enhances cholesterol accumulation in atherosclerotic plaques. In animal models, TMAO accumulation impairs liver function and increases hepatic triglyceride accumulation and lipogenesis, while in humans, TMAO increases levels of hepatic steatosis by regulating bile acid metabolism, increasing bile acid synthesis, and increasing the use of hepatic bile acids as an FXR antagonist. High TMAO levels are associated with vitamin D deficiency and nonalcoholic fatty liver disease (NAFLD) [3]. The abundance and composition of the gut microbiota have been proved that it could influence lipid metabolism in mouse and human blood and tissues [4]. In addition to CVD, TMAO has been linked to nonalcoholic fatty liver disease, diabetes, chronic kidney disease, and colorectal cancer [5]. TANG W H et al. have shown that TMAO is associated with a high risk of development of renal fibrosis and renal impairment, as well as reduced rates of long-term survival [6]. However, the mechanistic link between TMAO and diabetic kidney disease (DKD) remains to be explored.

In recent years, following the thorough study of the “intestinal-renal axis” and urinary toxins, TMAO has become the subject of a node of research activity in the field of nephropathy as a metabolite of intestinal bacteria and a urine toxin affecting the prognosis of CKD [7]. The roles of vitamin D/VDR receptor (VD/VDR) have been well documented in kidney protection. Studies have shown that the interaction between VD and gut microbes is closely related to inflammation [8, 9], VD favors probiotics rather than pathogenic microbial colonization when the body is in an inflammatory state [10, 11], and VDR activation exerts anti-inflammatory effects by inhibiting the activation of NF-κB in tubular and mesangial cells [12]. The association between decreased serum 25(OH)D levels in animal models of DKD and intestinal flora characteristics has been confirmed in many studies and in our previous studies [13, 14]. VD deficiency also mediates the expression of VDR in intestinal and renal tissues. However, it is unclear whether VD is involved in the changes in the intestinal flora seen in mice with DKD or in the relationship between TMAO, an intestinal bacteria metabolite, and the kidneys.

There are three types of fat around the kidneys: pararenal fat, antral fat, and perirenal adipose tissue (PRAT). PRAT is located in the retroperitoneal space and is considered as a simple connective tissue that protects the kidneys and renal vessels from external physical stimuli [15]. Because PRAT is anatomically in direct contact with the kidneys and adrenal glands, its presence can lead to various pathological abnormalities when body size increases (including expansion of perirenal adipose tissue) due to obesity or other problems (e.g., diabetes, DKD) [16]. Some scholars speculate that the expansion of perirenal adipose tissue means an increase in white adipocytes, which promote the progression of kidney damage [17]. BAT was previously thought to be absent in humans across all stages of life, from infancy to adulthood. However, with the development of devices measuring metabolic activity (fluoro-18-fluorodeoxyglucose positron emission tomography (18F-FDG-PET)/computed tomography (CT)), it has been found that a large amount of brown adipose tissue is found around the kidney and has high metabolic activity [16]. However, adipose tissue is considered as an endocrine organ that secretes various adipokines, rather than just storing energy. As an endocrine organ, PRAT contains many brown adipocytes and highly activated beige adipose cells produced by the transformation of white adipocytes [18]. The latter has the effect of increasing lipid catabolism and mitochondrial respiration, accelerating the energy expenditure of WAT, and reducing inflammation. Therefore, PRAT is considered a very useful source of cells in therapeutic terms.

Recent studies have shown that the production of the intestinal bacterial metabolite TMAO is associated with inhibition of the activities of the beige and white adipose tissue [19]. In addition, the TMAO synthesis pathway is becoming an increasingly attractive therapeutic target for obesity-related conditions such as type 2 diabetes, renal failure, and cardiovascular disease [20]. Typical markers of WAT browning are upregulation of uncoupling protein 1 (UCP1) expression and mitochondrial biosynthesis, a process that also enhances the cellular metabolism of adipocytes and the release of their metabolites. However, it is uncertain whether TMAO can promote inflammatory changes in perirenal adipocytes in mice with DKD and whether vitamin D plays a role in the effects of TMAO through VDR in the PRAT and the kidneys of mice with DKD. Therefore, this study explores the effects of the administration of different concentrations of 1,25-(OH)2D3 on the TLR4/NF-κB pathway in the intestinal flora and kidney tissue in mice with DKD, as well as the relationship between serum TMAO and urine ACR. Further, it explores whether TMAO has an effect on the metabolism of the adipose tissue around the kidney, in an intent to understand its effect on and possible relationship with active VD participation in the intestinal flora on the kidneys of mice with DKD. Finally, it proposes new insights for the prevention and treatment of DKD.

## Materials and methods

### Animals

Sixty 8-week-old male spontaneous KKay (type 2 diabetes) mice and 10 C57BL/6 mice (Beijing Huafukang Biotechnology Co., Ltd. (license number: SCXK (Beijing) 2020-0004)), all weighing 18–22 g, were raised in an SPF animal room, and the room temperature, humidity and indoor light/dark alternate time were standard.

KKay mice need High-sugar, high-fat and high-cholesterol feed (KK mice feed 1042). Ingredients: corn, soybean meal, wheat bran, wheat flour, sucrose, fish meal, egg yolk meal, soybean oil, lard, stone powder, dicalcium phosphate, salt, choline chloride, lysine, multivitamin, a variety of mineral elements. Nutritional analysis value: crude protein 17.5%, crude fat 17.9%, crude fiber 3.1%, crude ash 4.5%, moisture 8.5%, Calcium 0.88%, Total Phosphorus, 0.58%, and Nitrogen-free Extract, 48.5%.

### Methods

#### Experimental groups

Fifty-six KKay mice were divided into the DM group (*n* = 10, raised to 14 weeks of age) and DKD group (*n* = 46, raised to 20 weeks of age), and C57BL/6 mice were used as the control group (*n* = 10, raised to 20 weeks of age) randomly. The 56 mice in the DKD groups were divided into the placebo group (*n* = 10), low dosage (0.3 μg/kg/d) (*n* = 10), medium dosage (1.4 μg/kg/d) (*n* = 16), and high dosage (2.5 μg/kg/d) (*n* = 10) concentration 1,25-(OH)_2_D_3_ early intervention groups. The same volume of normal saline and the different concentrations of 1,25-(OH)_2_D_3_ were injected daily beginning when KKay mice reached 18 weeks of age for 14 days. Six of the DKD mice were administered 0.12% TMAO at the same time as medium-dose 1,25-(OH)2D3 injection and housed until 20 weeks of age. At the end of the experiment, mice were sacrificed for feces, urine, serum, perirenal adipose tissue and kidney tissue collection for subsequent experiments. This experimental setup was approved by the Laboratory Animal Welfare and Ethics Management Committee of Shanghai Veterinary Research Institute, Chinese Academy of Agricultural Sciences. (Ethics number:SV-20230908-07).

#### Weight, blood sugar and urine protein measurements

The weights of the mice were measured by a scale. Blood glucose was measured by tail clipping (blood glucose meter was purchased from Sannuo Biosensing Co., Ltd.). Urine samples of mice from each group were collected in metabolic cages, and the urine protein excretion rate, urine microalbumin content and urine creatinine content were detected by electrochemiluminescence method. Urine ACR = urine microalbumin/urine creatinine ratio (unit: mg/g). The urine tests were completed on the automatic biochemical analyzer (Beckman Coulter AU5821) The measurements are listed in Table 1.

**Table 1:** Differences between control and DKD mice.

	CON	DKD	*P* value
Glucose	7.32 ± 0.870	26.280 ± 2.931	<0.0001
Urine volume	2.2 ± 0.212	9.620 ± 0.864	<0.0001
Body weight (g)	26.34 ± 0.826	44.160 ± 2.707	<0.0001
Kidney weight (g)	0.246 ± 0.015	0.396 ± 0.050	0.0002
CR (umol/L)	21.86 ± 7.154	115.84 ± 27.691	<0.0001
UACR (mg/g)	12 ± 1.581	236.000 ± 47.101	<0.0001

#### Serum TNF-α and TMAO content determination

Ocular venous blood was taken and subjected to centrifugation, and serum TNF-α levels was detected by ELISA (R&D Systems, USA, MTA00B) and be read at 450 nm within 30 min. Serum TMAO was detected by LC‒MS assay under chromatographic conditions: ACQUITY UPLC® BEH HILIC Column (2.1 × 100 mm, 1.7 μm, Waters, USA).

#### Urine tubular injury molecule (KIM-1) and TMAO determination

Sandwich ELISA was used to detect urinary KIM-1 (Abcam, Inc., ab213477) and recoding at the OD at 450 nm. Urine TMAO detection was performed by LC‒MS assay, chromatographic conditions: ACQUITY UPLC® BEH HILIC Column (2.1 × 100 mm, 1.7 μm, Waters, USA).

#### Perirenal adipose tissue, kidney tissue and fecal specimen collection

Mice were sacrificed by cervical dislocation. The perirenal adipose tissue and kidneys were removed, and suspended in normal saline for later use. Approximately 0.5–1.0 g of feces from between the colon and rectum segments was collected and stored in a sterile cryopreservation tube in a freezer at −80 °C for subsequent experiments.

#### qPCR

qPCR was used to detect the mRNA expression of TLR4, NF-κB, PGC1α and UCP-1 in kidney tissue. TRIzol was used to extract total RNA, followed by reverse transcription and RT‒qPCR analysis. The 2^−△△CT^ method was used to calculate the relative total amount of the gene of interest. Primer sequences are listed in Table 2.

**Table 2:** PCR primer sequences.

Gene	Forward primer (5′–3′)	Reverse primer (3′–5′)
PGC1α	ATGGCATGGCTTACACCACC	GAGGCCAATTTTGTCTCCACA
UCP-1	ATGGCATGGCTTACACCACC	GAGGCCAATTTTGTCTCCACA
NF-κB	AGGCTTCTGGGCCTTATGTG	TGCTTCTCTCGCCAGGAATAC
MCP-1	TCTCTTCCTCCACCACTATGCA	GGCTGAGACAGCACGTGGAT
β-actin	GGCTGTATTCCCCTCCATCG	CCAGTTGGTAACAATGCCATGT

#### Fecal microbiota DNA extraction and variable region PCR amplification

The target sequence was selected after DNA was extracted from the feces from the colon and rectum, and the 16S rRNA V3-V4 region was amplified. The primers 338F (5′⁃ACTCCTACGGGAGGCAGCAGCA⁃3′)and806R (5′⁃GGACTACHVGGGTWTCTAAT⁃3′) were used for amplification. 16S rRNA was sequenced to determine the variety, quantity and function of the bacteria composing the intestinal flora.

#### Hematoxylin-eosin(H&E), Periodic Acid-Schiff (PAS), and Masson staining to observe the pathological changes in kidney tissue

H&E staining: The kidney tissue was fixed with 4% paraformaldehyde solution and paraffin-embedded. After 3–5 min of staining with hematoxylin, the cells were differentiated with 1% acid ethanol (1 ml of hydrochloric acid and 99 ml of 70% ethanol), immersed in running water, and then stained with eosin for 0.5–1 min before dehydration and sealing. The slides were observed under a light microscope (200× and 400×).

PAS staining: The samples were subjected to dewaxing, periodic acidification, water washes, light bleaching, color development, dehydration and sealing, followed by observation with a light microscope (200× and 400×).

Masson staining: Sample preparation included dewaxing, overnight incubation with potassium dichromate, hematoxylin staining, acid fuchsin staining, phosphomolybdenic acid treatment, aniline blue staining, dehydration and sealing, followed by observation under a light microscope (200× and 400×).

#### Immunofluorescence for podocin expression in the kidney

After tissue collection, PBS washing, 4% paraformaldehyde fixation, bovine serum blocking, 0.5% Triton X-100 membrane permeabilization treatment for 10 min, overnight 1:400 primary antibody incubation, 1 h 1:100 fluorescent secondary antibody incubation, PBS washing, anti-fluorescence quenching, mounting agent mounting, and fluorescence microscopy were performed. Each slide was photographed under a 10×/40× fluorescence microscope.

#### Immunohistochemistry to reveal the expression of VDR, PGC1α and UCP-1 in kidney and adipose tissue

Paraffin section dewaxing, antigen retrieval, blockade of endogenous peroxidase, serum blocking, primary antibody (VDR (ab3508) 1:2000; PGC1α (ab191838) 1:2000; UCP-1 (ab23430) 1:1000) overnight 4 °C incubation, 1 h 1:2000 secondary antibody incubation, PBS washing, DAB chromogen development, counterstaining of nuclei, dehydration and sealing were performed. Each slide was observed and photographed under a 10×/20× microscope. The hematoxylin-stained nucleus was blue, and the positive expression of DAB was brownish-yellow.

#### Electron microscopy to observe pathological changes in the kidneys and perirenal adipose tissue

Kidney and perirenal adipose tissue (1 mm^3^) was fixed in 2.5% glutaraldehyde for 4 h, fixed in 1% osmium acid for 1 h and then rinsed with PBS. Then, 2% uranium acetate water staining was performed for 30 min after dehydration, embedding and trimming. The cells were stained with 4% uranium acetate for 20 min, stained with lead citrate for 5 min, and then observed by transmission electron microscopy (10,000×). Three fields of view were taken from each mouse kidney and perirenal adipose tissue section, 10 areas of glomerular basement membrane thickness were randomly and continuously measured in each field of view, and the average value was taken to represent the thickness of the glomerular basement membrane.

#### Construction of a VDR knockout vector and VDR-KO mice

The VDR gene sequence (Gene ID: 22337) was determined by querying the NCBI database, and the CDS fragment of VDR was synthesized. The overexpression plasmid was cloned into a lentiviral vector. Firstly, three recombinant lentiviral vectors (a, b, c) targeting different VDR gene sequences were constructed, and then the virus silencing effect was verified by RT-PCR experiments, and the experiments showed that the effect of vector a was better. Then, the virus liquid a was injected into the mouse through the tail vein and then transfected into the kidney tissue of Kkay mice, and the green fluorescence distribution in the kidney tissue was seen by fluorescence imaging, indicating that Lenti-s hVDR transfection was effective and VDR-KO mice were constructed successfully.

### NovaSeq, bioinformatics analysis and statistical processing

NovaSeq Platform Sequencing (Paysenno Biomicrobiology LLC) was used to perform alpha and beta diversity and taxonomic composition analyses of the data. The analysis of between-group differences at the species and phylum levels was carried out using Metastats software.

SPSS 25.0 software and GraphPad Prism 8 were used for statistical analysis and graphing. Continuous variables were tested for a normal distribution, and continuous variables with a nonnormal distribution were logarithmically transformed to an approximately normal distribution. Measurement data are expressed as the mean ± standard deviation (SD). Independent sample t tests were used for comparisons between two groups, one-way analysis of variance (ANOVA) was used for comparisons between three groups or more, and the LSD test was used for two-by-two comparisons of multi-sample means. The Mann‒Whitney U test was used for determining bacterial taxonomic differences, Pearson correlation analysis was used for correlation analysis, and *P* < 0.05 indicated that the differences were statistically significant.

## Results

### Construction of DKD experimental mouse model

**Fig. 1:**
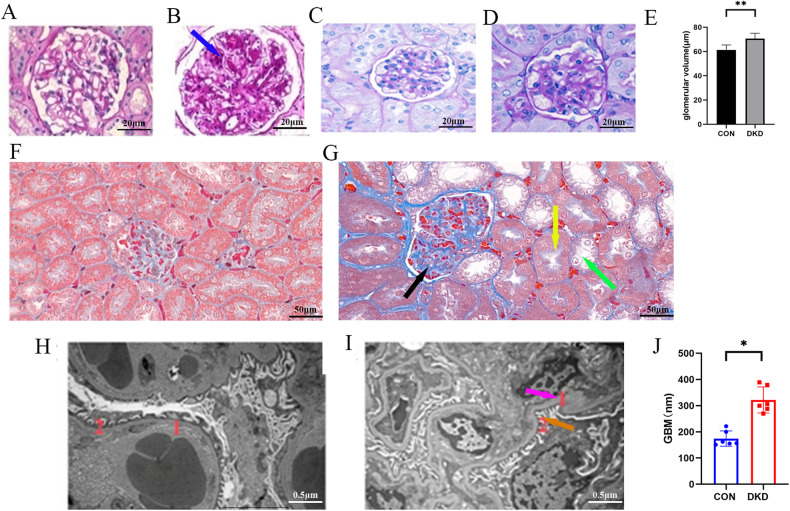
Establishment of the DKD experimental mouse model. **A** HE staining of the glomerulus in the control group (400×). **B** HE staining of the glomerulus in the DKD group (400×). **C** PAS staining of the glomerulus in the control group (400×). **D** PAS staining of the glomerulus in the DKD group (400×) **E** Glomerular volume in CON and DKD group. **F** Masson staining of the renal cortex in the CON group (400×). **G** Masson staining of the renal cortex in the DKD group (400×). **H** Electron microscopy of the kidney in the control group (10000×). **I** Electron microscopy of the kidney in the DKD group (10000×) (1: Basement membrane; 2: foot processes). **J** Thickness of the glomerular basement membrane in the CON and DKD group.

Mice were administered high-fat and high-sugar feed until they reached 20 weeks of age. As the mice aged, their body weights and blood glucose levels increased. In DKD group, the level of average blood glucose level, urine volume, kidney weight, body weight, creatinine (CR), urine albumin creatine ratio (UACR) increased. (see Table 2) Besides, diffuse thickening of the glomerular basement membrane (GBM) (purple arrows, Con: 174.5 ± 29.15 nm; DKD: 322 ± 49.89 nm; see Fig. 1I, J), segmental foot fusion (brown arrows; see Fig. 1I), KW nodules (blue arrows), tubular vacuolization (green arrows), epithelial edema (yellow arrows), interstitial fibrosis (black arrows) and other pathological changes (see Fig. 1A–D, F, G) confirmed that the DKD model was successfully established.

### Effects of vitamin D supplementation on renal function, intestinal flora characteristics, TMAO metabolites, and the TLR4/NF-κB pathway in mice with DKD

#### Effect of different doses of vitamin D on the kidney

**Fig. 2:**
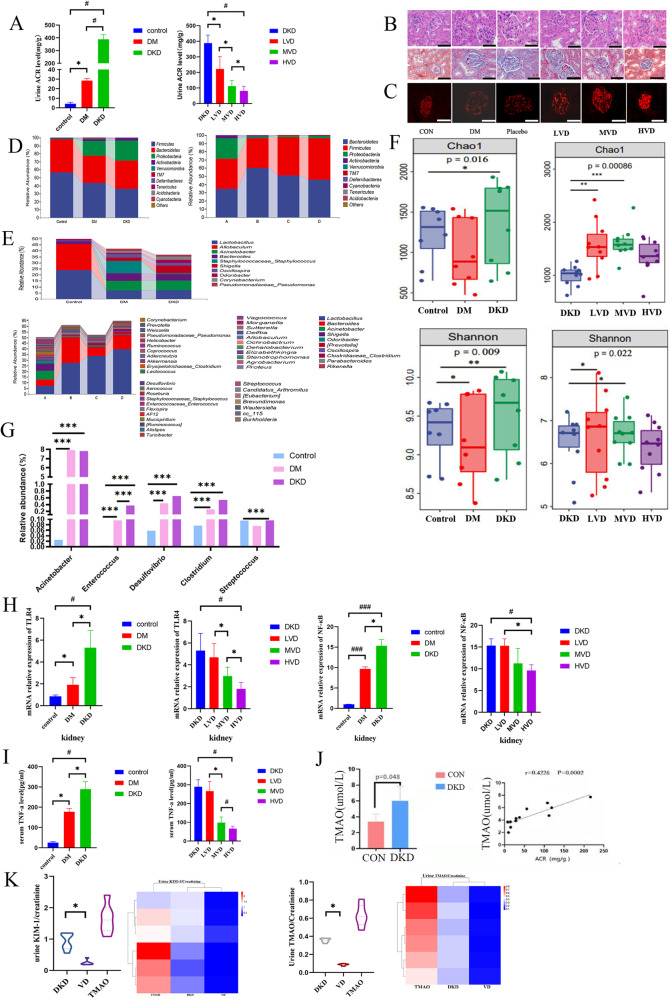
Effects of vitamin D supplementation on renal function, intestinal flora characteristics, TMAO metabolites, and the TLR4/NF-κB pathway in mice with DKD. **A** Urine ACR results (*n* = 6 mice per group). DKD diabetic kidney disease, LVD low-dose vitamin D group, MVD medium-dose vitamin D group, HVD high-dose vitamin D group *p < 0.05, #*p* < 0.01; **B** HE, Masson staining. **C** Immunofluorescence staining (with an anti-podocin antibody) of kidney sections; 18 images per 6 biological replicates for each group, 200×. **D** Histogram of the relative abundance of the top 10 bacteria at the phylum level (*n* = 6 mice per group). **E** Histogram of the relative abundance of bacteria at the genus level (*n* = 6 mice per group). **F** Shannon and Chao1 indices. **G** Histogram of TMAO-producing bacteria (*n* = 6 mice per group). **H** Quantitative relative mRNA expression of TLR4 and NF-κB (*n* = 6 mice per group). *p < 0.05, #p < 0.01, ###*p* < 0.001. **I** Serum TNF-α concentration (*n* = 6 mice per group). **J** Analysis of serum TMAO concentration and correlation with urine ACR (*n* = 6 mice per group). **K** Serum of TMAO, urine KIM-1, and urine TMAO/creatinine concentration (*n* = 6 mice per group).

The results of urine protein analysis showed that the urine ACR of mice in the DKD group was significantly greater than that of DM mice and control mice. After 1,25-(OH)2D3 supplementation, the mental state and diet of the mice improved. The concentration of 1,25-(OH)2D3 was defined as high (HVD, 2.5 μg/kg/d), low (LVD, 0.3 μg/kg/d) or medium (MVD, 1.4 μg/kg/d) concentrations. With an increase in the concentration of 1,25-(OH)2D3, the urine protein content began to decrease, and the HVD group had the most substantial decrease compared to the other groups. The concentration dependence of 1,25-(OH)2D3 was significant (Fig. 2A). Pathology by HE and Masson staining revealed that the glomerular volume of mice with DKD increased with the incidence of mesangial stromal hyperplasia, and the glomerulus and tubular and interstitial fibrotic lesions changed. The sizes of the glomerular, tubular and interstitial fibrotic lesions were reduced with the administration of different concentrations of 1,25-(OH)2D3, and compared with those in the MVD and LVD groups, the size of the lesions in the HVD group was significantly reduced (Fig. 2B). Immunofluorescence observation of renal podocin expression showed that the glomerular volume in mice in the DM and DKD groups increased, podocin expression decreased, and podocin expression in kidney tissues increased with the administration of different concentrations of 1,25-(OH)2D3. These parameters were significantly improved in the HVD group compared with those in the MVD and LVD groups (Fig. 2C).

#### Fecal microbiome of mice in the control, DM and DKD groups

The results of NovaSeq and bioinformatics analysis suggested that the abundance of *Bacillota* in the gut microbiota of the three groups of mice was 56.46% in the control group, 43.25% in the DM group and 36.38% in the DKD group; the abundance of *Bacteroidota* was 41.62% in the control group, 34.31% in the DM group and 34.89% in the DKD group; and the proportion of *Pseudomonadota* in the control group was very low, while that in the DM and DKD groups increased by 18.27% and 25.22%, respectively (Fig. 2D). At the genus level, *Proteus, Lactobacillus, Staphylococcus* and *Shigella* were the main genera. The control group had a greater relative abundance of *Lactobacillus* and other bacteria (*P* = 0.001), while the DKD group had a relatively high abundance of Shigella and other bacteria (*P* = 0.001). After 1,25-(OH)2D3 intervention, the richness index of the gut microbiota increased significantly, indicating that the diversity of the intestinal flora increased after 1,25-(OH)2D3 supplementation, and the high abundance of G- bacteria in the DKD group decreased after 1,25-(OH)2D3 intervention (Fig. 2F). There was a significant difference in the gut microbiota composition of DM and DKD mice. The richness and diversity of the gut microbiota in DKD mice were increased and in DM mice were reduced (Fig. 2F); the proportions of intestinal bacteria associated with TMAO production, such as *Desulfovibrio, Clostridium, Enterococcus, Streptococcus* and *Acinetobacter*, were significantly greater in DKD and DM mice than in the control groups (Fig. 2G) but for *Enterococcus*, the proportion in DKD mice was higher than in DM mice.

#### The effect of vitamin D and TMAO on inflammatory pathways

Compared with those in the control group, the expression of TLR4 and NF-κB mRNA in kidney tissues in the DM and DKD groups was upregulated, and that in the DKD group was greater than that in the DM group. After 1,25-(OH)2D3 intervention, the mRNA expression of TLR4 and NF-κB in kidney tissues was downregulated, and the HVD group exhibited significant improvement compared with the MVD and LVD groups (Fig. 2H). Compared with those in the control group, TNF-α concentrations were increased in both the DM and DKD groups (*P* = 0.000). Compared with that in the DKD group, the serum TNF-α concentration in DKD mice decreased significantly after 1,25-(OH)2D3 treatment, and the serum TNF-α concentration showed a dose‒response relationship with increasing 1,25-(OH)2D3 concentrations; the difference was significant (*P* = 0.001) (Fig. 2I). The serum TMAO concentration in DKD mice was significantly greater than that in control mice, and the serum TMAO concentration was positively correlated with the urine ACR concentration (r = 0.4226, *P* = 0.0002) (Fig. 2J). The serum and urine TMAO concentrations were significantly lower after vitamin D treatment, and expression of the renal injury marker KIM-1 was significantly downregulated (Fig. 2K).

Previous studies by our group found that low, medium and high concentrations of 1,25-(OH)_2_D_3_ had an effect of improving kidney damage in DKD, although the HVD group improved more significantly than the MVD and LVD groups. However, we observed that although the effect of high-concentration 1,25-(OH)_2_D_3_ intervention was better than that of low and medium concentrations, 3 mice died after high-concentration 1,25-(OH)_2_D_3_ intervention. Hypoglycemia occurred the day before death, and the measured blood glucose levels of the mice were 2.2, 2.8, and <1.1 mmol/L, respectively. The specific reason for the deaths is unknown, so a medium concentration (1.4 μg/kg/d) of 1,25-(OH)_2_D_3_ was used for intervention for subsequent experiments.

### VD played a role in the effect of TMAO on perirenal fat and kidney in mice with DKD

**Fig. 3:**
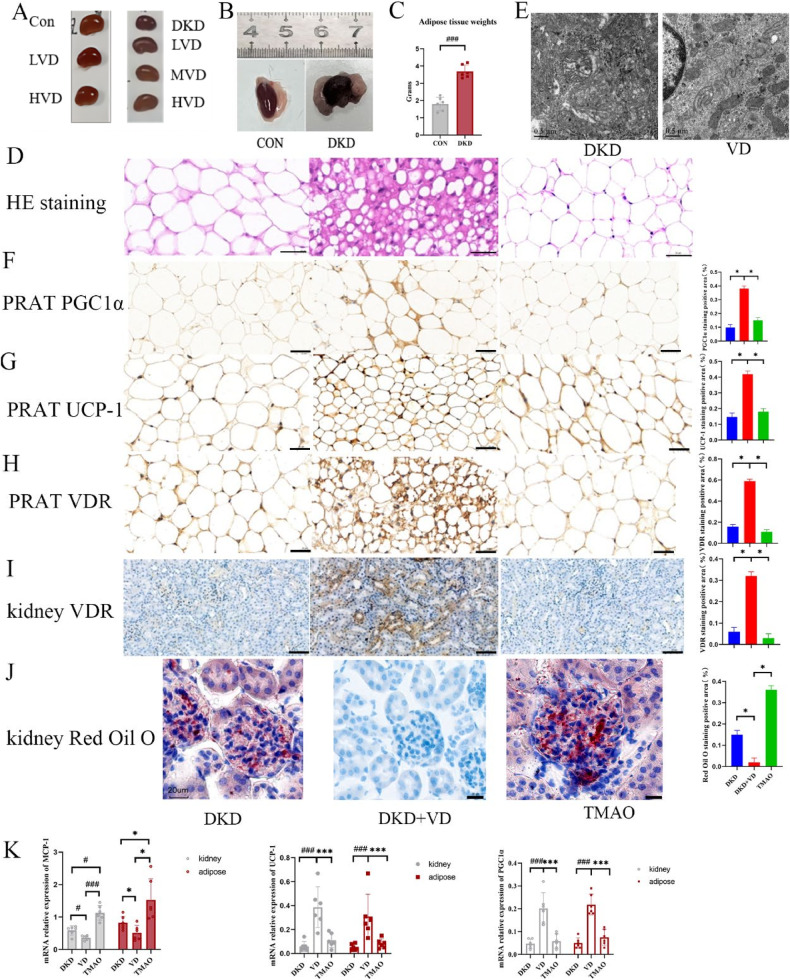
VD played a role in the effect of TMAO on perirenal fat and kidney in mice with DKD. **A** kidney tissue of different groups after 1,25-(OH)2D3 intervention. **B** Gross anatomy of perirenal adipose tissue. **C** Comparison of fat mass (*n* = 6 mice per group). **D** HE staining for Perirenal adipose tissue (18 images per 6 biological replicates for each group). Scale bar, 200×. **E** Electron microscopy for Perirenal adipose tissue in DKD and1,25-(OH)2D3 intervention group(VD) (*n* = 6 mice per group). Red arrow: mitochondria, blue arrow: lipid droplets, N: nucleus. Scale bar, 100 μm. (25,000×). **F**–**I** IHC-stained (with an anti-PGC1α, UCP1 and VDR antibody) (18 images per 6 biological replicates for each group) sections of PPAT and kidney. Scale bar, 50μm. (200×). **J** Oil stained (18 images per 6 biological replicates for each group). (400×). **K** Kidney and PRAT MCP-1, PGC1α and UCP-1 mRNA expression (1 technical replicate of 6 biological replicates per group).

We collected kidney and perirenal adipose tissue samples from DKD mice (Fig. 3A, B) and found that the kidneys of DKD mice were surrounded by a layer of beige adipose tissue, and the fat mass of DKD mice was significantly higher than the control group (Fig. 3C). These samples were stained with hematoxylin and eosin (H&E) (Fig. 3D) and perirenal fat appeared to shift towards a beige adipose tissue phenotype after vitamin D intervention, revealing multilocular adipocytes of various sizes. Electron microscopy (Fig. 3E) showed abundant mitochondria, mitochondrial swelling, crest fracture loss, and plate layer reduction in perirenal adipocytes in the DKD group; the expression of perirenal fat and renal VDR increased and the mitochondrial structure of adipocytes was significantly improved after vitamin D treatment. Compared with the control group, DKD mice fed a high-fat diet had more adipose cells and higher levels of hypertrophy. In DKD mice, intravenous injection with 1.4 μg/kg/d 1,25-(OH)_2_D_3_ and dietary supplementation with 0.12% TMAO caused a shift towards beige adipose tissue in the 1,25-(OH)_2_D_3_ intervention group, indicating that 1,25-(OH)_2_D_3_ could effectively improve adipocyte hypertrophy. Adipose cells were more hypertrophied after TMAO administration. We performed histochemical and qPCR experiments, and the results showed that VDR expression decreased in the DKD group, and the expression of PGC1α (Fig. 3F) and UCP-1 (Fig. 3G) were increased after 1,25-(OH)_2_D_3_ intervention, in both the kidney and PRAT beige adipocytes, with good reproducibility. Vitamin D stimulated upregulated expression of VDR, while PRAT and renal VDR expression were downregulated after TMAO supplementation (Fig. 3H, I). Oil red O staining showed a significant increase in PRAT lipid infiltration after TMAO intervention and an effective reduction in PRAT lipid infiltration after 1,25-(OH)_2_D_3_ intervention (Fig. 3J), as well as lowered concentration of the inflammatory cytokine MCP-1 (Fig. 3K).

### Inhibition of VDR expression affects the function of glomerular podocytes in DKD mice

**Fig. 4:**
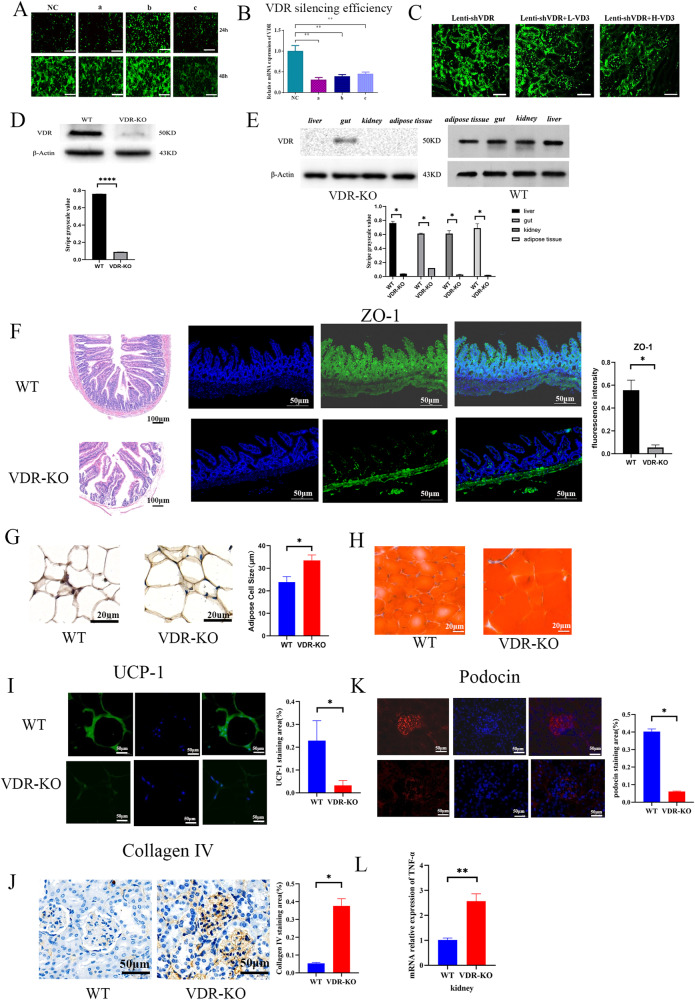
Inhibition of VDR expression affects the function of glomerular podocytes in DKD patients. **A** Fluorescence imaging of three different concentrations of viral fluid in 293T cells. Fluorescence imaging after 24 h transfer of 293T cells (upper layer, 100×); Fluorescence imaging after 48 h transfer of 293T cells (lower layer, 200×). NC: Blank control, no viral fluid injection; a: Viral fluid with an average titer of 3.02E + 08; b: Viral fluid with an average titer of 2.60E + 08; c: RT-PCR verification of 3 viral fluids with an average titer of 2.81E + 08. **B** RT‒PCR verification of different titer for VDR silencing efficiency. **C** renal tissue recombinant lentiviral immunofluorescence validation (×200). **D** VDR protein expression (*n* = 6 mice per group). **E** representative images of the liver, intestine, kidney, adipose tissue, and VDR protein expression. (*n* = 6 mice per group). **F** intestinal HE staining (×100) and ZO-1 fluorescence (×200). The intestinal mucosa is destroyed and permeability increases. **G** Representative immunohistochemistry image of VDR expression. (×400). **H** Oil stained. **I** Representative image of UCP-1 immunofluorescence in the kidney (×400). (*n* = 6 mice per group). **J** Representative image of renal collagen IV histochemistry (×400). **K** Representative image of kidney podocin immunofluorescence (×400) (*n* = 6 mice per group) (*n* = 6 mice per group). **L** TNF-α expression; *p* < 0.05 (*n* = 6 mice per group).

293T cells were transfected with a recombinant lentiviral vector containing the sequence of the 3.02E + 08 mouse VDR gene. Transfection for 48 h induced effective VDR knockdown (see Fig. 4A). Fluorescence imaging of Kkay mouse kidney tissue transfected with viral fluid through the tail vein showed green fluorescence distribution in kidney tissue, indicating that Lenti-shVDR transfection was effective (see Fig. 4C). VDR-KO mouse renal VDR expression was significantly lower than that of the control group as determined by WB (see Fig. 4D). VDR expression was reduced in liver, intestine, kidney, and adipose tissue (see Fig. 4E). The intestinal mucosa was disrupted, and permeability was increased (see Fig. 4F). In PRAT, single cells were enlarged in the VDR-KO group, and the fat content increased at the same volume (see Fig. 4G). The amount of PRAT did not change, and levels of indicators of energy metabolism and brown fat decreased (see Fig. 4I). Inhibition of VDR expression affected the function of DKD glomerular podocytes, which were in an inflammatory state. The expression of TNF-α was also increased (see Fig. 4L), the expression of podocin protein decreased (see Fig. 4K), and the expression of the fibrosis indicator collagen IV increased (see Fig. 4J).

## Discussion

In 2007, the National Kidney Foundation (NKF) developed guidelines for improved quality of life in patients with kidney disease, recommending the replacement of the term “diabetic nephropathy” (DN) with DKD [21]. DKD is a serious chronic renal complication of DM and a leading cause of death in patients with DM and of renal failure in patients with ESRD [22].

With the development of sequencing technology, the intestinal flora has attracted much attention in recent years. The intestinal flora is strongly associated with many chronic diseases [23, 24], and *Ackermannia* species are abundant in patients with type 2 diabetes [25]. Studies have shown that SCFA-producing bacteria and Megasphaera in patients with T2DM and DKD may be associated with the development of DKD, while harmful bacteria such as *Escherichia* and *Enterococcus* are more abundant in patients with DKD than in healthy people. In this study, 16S RNA sequencing showed changes in the abundance of gut microbiota constituents in DKD mice. Specifically, the abundance of beneficial bacteria, such as lactic acid bacteria, decreased, while the abundance of G bacteria increased significantly. However, to date, the effect of vitamin D (VD) on the intestinal flora in patients with DKD has rarely been reported.

In recent years, the protective effect of VD and the vitamin D receptor (VDR) in the kidney has been well demonstrated, but its underlying mechanism is unclear [26]. VD deficiency has been shown to be an important risk factor for the onset of chronic kidney disease (CKD), and aggressive supplementation with VD can improve survival in patients with CKD [27]. Studies have shown that the interaction between VD and gut microbes may be closely related to the regulation of inflammation [8, 9], but the specific mechanism involved is not clear. We have previously confirmed that VD directly exerts a protective effect on kidneys in a DKD model, but whether it also has an effect on perirenal adipose tissue (PRAT), intestinal flora and trimethylamine oxide (TMAO) may be related.

W. H. Wilson Tang et al. suggested that the gut microbiota relies on the TMAO synthesis pathway to promote the development of renal insufficiency and to increase the risk of death in chronic kidney disease [6]. TMAO inhibits macroprotein expression and albumin uptake in human proximal tubular cells through PI3K and ERK signaling [28] but its effect on glomeruli is unclear. Previous studies have shown that a variety of intestinal bacteria, such as *Enterobacteriaceae, Escherichia, Desulfuros, Clostridium, Enterococcus*, and *Actinomycetes*, are involved in the production of TMAO metabolites. FENNEMA et al. [5] summarized the types of intestinal bacteria involved in TMA production, among which intestinal actinomycetes (*Kinymoflex, Eurlosenia)*, Helicobacter (*Clostridium, Enterococcus, Streptococcus*), proteobacteria (*Vibrio desulfuris, Edwards, Enterobacteriaceae, Escherichia*) and other strains can encode the catalytic enzymes required to degrade choline and promote the conversion of choline to TMA.

Our previous studies revealed that vitamin D has a protective effect on diabetic nephropathy, and at the same time, studies have shown that increasing dietary vitamin D intake affects gut microbial regulation and improves intestinal barrier function by stabilizing tight junctions between the epithelium, thereby promoting the balance between the microbiota and intestinal immunity. This balance plays a key role in maintaining immune homeostasis in healthy populations [29]. Therefore, in this study, we explored whether the effect of vitamin D on the gut microbiota indirectly affects kidney function through the gut-kidney axis and the relationship between vitamin D and TMAO.

Previous studies by our group revealed that low, medium and high concentrations of 1,25-(OH)2D3 reduced kidney damage in DKD patients, although the HVD group showed more improvement than the MVD and LVD groups. However, although the effect of high-concentration 1,25-(OH)2D3 intervention was greater than that of the low- and medium-concentration 1,25-(OH)2D3 interventions, 3 mice died after high-concentration 1,25-(OH)2D3 intervention. Hypoglycemia occurred the day before death, and the measured blood glucose levels were 2.2, 2.8, and <1.1 mmol/L. The specific reason for the deaths was unknown, so a medium concentration (1.4 μg/kg/d) of 1,25-(OH)2D3 was used for intervention in subsequent experiments. In our experiments, the serum TMAO concentration in DKD mice was significantly greater than that in control mice, and the serum TMAO concentration was positively correlated with urinary ACR, thus indicating that TMAO exacerbates renal insufficiency. KIM-1, a marker of kidney injury in mice with diabetic nephropathy treated with vitamin D, was significantly reduced, and the level was significantly increased after TMAO intervention, suggesting that vitamin D can reduce kidney injury and that TMAO can antagonize this protective mechanism. Moreover, we measured inflammatory factor levels in the gut and kidneys after TMAO intervention and found that they were significantly elevated.

Therefore, we hypothesized that these harmful and beneficial bacteria activate cytokines and induce systemic inflammatory responses by regulating the secretion of the intestinal metabolite TMAO. These intestinal metabolites are likely directly involved in or exacerbate the pathogenesis of DKD, and vitamin D can antagonize this effect.

To explore the antagonistic effect of vitamin D on the intestinal microbiota and its possible mechanism, we investigated the intestinal microbiota of diabetic mice and diabetic nephropathy mice after vitamin D intervention and found that the gut microbiota richness index increased significantly after 1,25-(OH)_2_D_3_ intervention, indicating that the diversity of the gut microbiota increased after 1,25-(OH)_2_D_3_ supplementation and the abundance of Firmicutes gradually increased with increasing 1,25-(OH)_2_D_3_ concentration. These findings are similar to other recent findings [10, 11, 30, 31]. The effect of 1,25-(OH)_2_D_3_ on the gut microbiota is inconclusive, but our study is consistent with the findings of Bashir et al., who revealed that the relative abundance of *Proteus* decreased and that the relative abundance of *Bacteroides* increased after VD supplementation. However, their study involved sequencing intestinal mucosal tissue [32]. Studies have reported that VD signaling favors probiotic colonization when the body is in an inflammatory state, and VD supplementation can activate VDR and exert anti-inflammatory effects by inhibiting NF-κB activation in tubular and mesangial cells [12].

In this study, we also revealed that with increasing expression of TLR4/NF-κB inflammatory signaling pathway markers in the kidney tissues of DM and DKD mice, the concentration of the inflammatory cytokine TNF-α and the urine protein levels increased; there was also exacerbation of pathological changes, such as glomerular hypertrophy, renal glomeruli and interstitial fibrosis, tubulointerstitial widening, fibrosis, epithelial edema and vacuolation. Moreover, glomerular podocytes exhibited decreased expression of podocin. Compared with those in the control group, the expression of urine proteins and TLR4/NF-κB signaling pathway markers was downregulated, the expression of podocin was increased, and the inflammatory state and pathological changes were improved after 1,25-(OH)_2_D_3_ intervention. Further experiments in which VDR was knocked out verified that the inhibition of VDR expression affects the function of glomerular podocytes in patients with diabetic nephropathy and that the expression of podocin is reduced during the inflammatory state in podocytes. These experimental results show that vitamin D can reduce inflammation by directly acting on the kidneys, thereby protecting the kidneys

Adipose tissue is an important storage site for VD. VD deficiency is associated with abnormal fat production. This study found that VD supplementation improved renal fat infiltration in mice with DKD.

Studies have also shown that increasing dietary vitamin D intake affects intestinal microbial regulation and increases intestinal barrier function by stabilizing tight junctions between the epithelia, thereby promoting the balance between microbiota and intestinal immunity, which plays a key role in maintaining immune homeostasis in healthy people [10, 29].

Obeid et al. reported that vitamin D supplementation for 12 months is effective in reducing plasma fasting TMAO content and increasing plasma choline, a precursor to TMAO and an essential component for synthesizing phosphatidylcholine, which in turn is necessary to promote lipid output from the liver [33]. SHERRIFF J L speculates that the deficiency of choline, a dietary nutrient, is associated with the accumulation of lipids in the liver [34]. It has been suggested that low vitamin D levels are associated with high TMAO levels and changes in the gut microbiota in obese individuals, as well as with the severity of NAFLD [3, 7]. A cross-sectional study found that vitamin D levels decreased significantly with increasing plasma TMAO concentrations; vitamin D levels were lowest and TMAO levels were highest in individuals with class III obesity [3]. Most studies to date have linked high TMAO levels to vitamin D deficiency and NAFLD. There is a strong link between the TMA/FMO3/TMAO pathway and adipose tissue function [19]. All of the above studies suggest that vitamin D levels show a significant negative correlation with circulating TMAO levels. Our study partially explains that the mechanism of this phenomenon may be related to TMAO promoting fat deposition and vitamin D reducing fat deposition.

The presence of perirenal adipose tissue (PRAT) has recently been identified as an independent risk factor for CKD progression and is associated with cardiac and renal dysfunction. Due to the peculiarities of its anatomical location, PRAT protects the kidneys and renal vessels from external physical stimuli. PRAT is adjacent to the kidneys, and they share innervation and circulation. PRAT dilation, dysfunction, and inflammation are thought to have a significant impact on kidney function. However, it is unknown whether there is a relationship between the perirenal fat microenvironment and kidney damage in diabetic nephropathy (DKD). In this study, we found that the lipid infiltration of PRAT increased significantly after TMAO intervention, PGC1α and UCP-1 expression increased after vitamin D intervention in both the kidney and in PRAT, and the concentration of the inflammatory cytokine MCP-1 was decreased. Comparing DKD mouse PRAT with ordinary mouse PRAT, we found that the volume of PRAT in DKD mice was significantly increased, and we hypothesized that TMAO can act on PRAT and thereby have an impact on kidney function, while vitamin D can antagonize the effect of TMAO.

However, this study did not thoroughly study the changes in intestinal and renal pathology or the changes in intestinal flora after supplementation with probiotics or fecal bacteria transplantation. This study also did not thoroughly explore the antagonistic mechanism associated with vitamin D and TMAO, which must be explored in depth in the future.

In summary, intestinal dysbacteriosis and its metabolite TMAO may be involved in the pathogenesis of DKD, and the protective effect of 1,25-(OH)2D3 on kidney tissue in DKD mice may be related to the inhibition of the activation of the renal TLR4/NF-κB inflammatory signaling pathway after improving intestinal dysbacteriosis. TMAO can affect PRAT and kidney function, but the effect of the microflora and its metabolites on kidney tissue and PRAT and the specific mechanism of action between kidney and PRAT in DKD needs to be further studied.

## Data Availability

The datasets generated during and/or analyzed during the current study are available from the corresponding author on reasonable request.

## References

[1] Olofsson LE, Backhed F (2022). The Metabolic Role and Therapeutic Potential of the Microbiome. Endocr Rev.

[2] Koeth RA, Wang Z, Levison BS, Buffa JA, Org E, Sheehy BT (2013). Intestinal microbiota metabolism of L-carnitine, a nutrient in red meat, promotes atherosclerosis. Nat Med.

[3] Barrea L, Muscogiuri G, Annunziata G, Laudisio D, De Alteriis G, Tenore GC, et al. A New Light on Vitamin D in Obesity: A Novel Association with Trimethylamine-N-Oxide (TMAO). Nutrients. 2019;11:1310. 10.3390/nu11061310 PMC6627576 31185686

[4] Schoeler M, Caesar R (2019). Dietary lipids, gut microbiota and lipid metabolism. Rev Endocr Metab Disord.

[5] Fennema D, Phillips IR, Shephard EA (2016). Trimethylamine and Trimethylamine N-Oxide, a Flavin-Containing Monooxygenase 3 (FMO3)-Mediated Host-Microbiome Metabolic Axis Implicated in Health and Disease. Drug Metab Dispos.

[6] Tang WH, Wang Z, Kennedy DJ, Wu Y, Buffa JA, Agatisa-Boyle B (2015). Gut microbiota-dependent trimethylamine N-oxide (TMAO) pathway contributes to both development of renal insufficiency and mortality risk in chronic kidney disease. Circ Res.

[7] Zhao L, Lou H, Peng Y, Chen S, Zhang Y, Li X (2019). Comprehensive relationships between gut microbiome and faecal metabolome in individuals with type 2 diabetes and its complications. Endocrine.

[8] Barengolts E (2013). Vitamin D and prebiotics may benefit the intestinal microbacteria and improve glucose homeostasis in prediabetes and type 2 diabetes. Endocr Pr.

[9] Cantorna MT, Lin YD, Arora J, Bora S, Tian Y, Nichols RG (2019). Vitamin D Regulates the Microbiota to Control the Numbers of RORgammat/FoxP3+ Regulatory T Cells in the Colon. Front Immunol.

[10] Ciubotaru I, Green SJ, Kukreja S, Barengolts E (2015). Significant differences in fecal microbiota are associated with various stages of glucose tolerance in African American male veterans. Transl Res.

[11] Jahani R, Fielding KA, Chen J, Villa CR, Castelli LM, Ward WE (2014). Low vitamin D status throughout life results in an inflammatory prone status but does not alter bone mineral or strength in healthy 3-month-old CD-1 male mice. Mol Nutr Food Res.

[12] Yang S, Li A, Wang J, Liu J, Han Y, Zhang W (2018). Vitamin D Receptor: A Novel Therapeutic Target for Kidney Diseases. Curr Med Chem.

[13] Nakhoul N, Thawko T, Farber E, Dahan I, Tadmor H, Nakhoul R (2020). The Therapeutic Effect of Active Vitamin D Supplementation in Preventing the Progression of Diabetic Nephropathy in a Diabetic Mouse Model. J Diabetes Res.

[14] Zhao WJ, Xia XY, Yin J (2021). Relationship of serum vitamin D levels with diabetic microvascular complications in patients with type 2 diabetes mellitus. Chin Med J.

[15] Okhunov Z, Mues AC, Kline M, Haramis G, Xu B, Mirabile G (2012). Evaluation of perirenal fat as a predictor of cT 1a renal cortical neoplasm histopathology and surgical outcomes. J Endourol.

[16] Zhu Q, Scherer PE (2018). Immunologic and endocrine functions of adipose tissue: implications for kidney disease. Nat Rev Nephrol.

[17] Grigoraș A, Balan RA, Căruntu ID, Giușcă SE, Lozneanu L, Avadanei RE, et al. Perirenal Adipose Tissue-Current Knowledge and Future Opportunities. J Clin Med. 2021;10:1291. 10.3390/jcm10061291 PMC8004049 33800984

[18] Wei G, Sun H, Dong K, Hu L, Wang Q, Zhuang Q (2021). The thermogenic activity of adjacent adipocytes fuels the progression of ccRCC and compromises anti-tumor therapeutic efficacy. Cell Metab.

[19] Schugar RC, Shih DM, Warrier M, Helsley RN, Burrows A, Ferguson D (2017). The TMAO-Producing Enzyme Flavin-Containing Monooxygenase 3 Regulates Obesity and the Beiging of White Adipose Tissue. Cell Rep.

[20] Schugar RC, Willard B, Wang Z, Brown JM (2018). Postprandial gut microbiota-driven choline metabolism links dietary cues to adipose tissue dysfunction. Adipocyte.

[21] KDOQI. (2007). KDOQI Clinical Practice Guidelines and Clinical Practice Recommendations for Diabetes and Chronic Kidney Disease. Am J Kidney Dis.

[22] Sharma D, Bhattacharya P, Kalia K, Tiwari V (2017). Diabetic nephropathy: New insights into established therapeutic paradigms and novel molecular targets. Diabetes Res Clin Pr.

[23] Chen Y, Zhou J, Wang L (2021). Role and Mechanism of Gut Microbiota in Human Disease. Front Cell Infect Microbiol.

[24] Hills RD Jr, Pontefract BA, Mishcon HR, Black CA, Sutton SC, Theberge CR. Gut Microbiome: Profound Implications for Diet and Disease. Nutrients. 2019;11:1613. 10.3390/nu11071613 PMC6682904 31315227

[25] Fan Y, Pedersen O (2021). Gut microbiota in human metabolic health and disease. Nat Rev Microbiol.

[26] Jiang BT, Chen QZ, Guo ZH, Zou W, Chen X, Zha WL (2016). Ischemic post-conditioning attenuates renal ischemic reperfusion injury via down-regulation of toll-like receptor 4 in diabetic rats. Ren Fail.

[27] Christakos S, Dhawan P, Verstuyf A, Verlinden L, Carmeliet G (2016). Vitamin D: Metabolism, Molecular Mechanism of Action, and Pleiotropic Effects. Physiol Rev.

[28] Christakos S, Dhawan P, Verstuyf A, Verlinden L, Carmeliet G. TMAO Suppresses Megalin Expression and Albumin Uptake in Human Proximal Tubular Cells Via PI3K and ERK Signaling. Int J Mol Sci. 2022;23:8856. 10.3390/ijms23168856 PMC9407713 36012119

[29] Derakhshanian H, Shab-Bidar S, Speakman JR, Nadimi H, Djafarian K (2015). Vitamin D and diabetic nephropathy: A systematic review and meta-analysis. Nutrition.

[30] Urushihara M, Kagami S (2017). Role of the intrarenal renin-angiotensin system in the progression of renal disease. Pediatr Nephrol.

[31] Yuan Y, Sun H, Sun Z (2017). Advanced glycation end products (AGEs) increase renal lipid accumulation: a pathogenic factor of diabetic nephropathy (DN). Lipids Health Dis.

[32] Rinninella E, Raoul P, Cintoni M, Franceschi F, Miggiano GAD, Gasbarrini A, et al. What is the Healthy Gut Microbiota Composition? A Changing Ecosystem across Age, Environment, Diet, and Diseases. Microorganisms. 2019;7:14. 10.3390/microorganisms7010014 PMC6351938 30634578

[33] Obeid R, Awwad HM, Kirsch SH, Waldura C, Herrmann W, Graeber S, et al. Plasma trimethylamine-N-oxide following supplementation with vitamin D or D plus B vitamins. Mol Nutr Food Res. 2017;61. 10.1002/mnfr.201600358 27569255

[34] Sherriff JL, O’sullivan TA, Properzi C, Oddo JL, Adams LA (2016). Choline. Its Potential Role in Nonalcoholic Fatty Liver Disease, and the Case for Human and Bacterial Genes. Adv Nutr.

